# The Value of Nature During Psychotherapy: A Qualitative Study of Client Experiences

**DOI:** 10.3389/fpsyg.2021.765177

**Published:** 2021-11-10

**Authors:** Daphne Meuwese, Nienke van der Voort, Karin Dijkstra, Lydia Krabbendam, Jolanda Maas

**Affiliations:** ^1^Department of Clinical, Neuro and Developmental Psychology, Vrije Universiteit Amsterdam, Amsterdam, Netherlands; ^2^Altrecht Institute for Mental Health Care, Utrecht, Netherlands; ^3^Research Group Nursing, Saxion University of Applied Sciences, Enschede, Netherlands

**Keywords:** psychotherapy, restorative environments, mental health, clinical psychology, nature

## Abstract

Nature is considered to have restorative qualities that can potentially improve psychotherapy success. However, little is known about how clients experience nature during psychotherapy. The research aim of this phenomenological qualitative study was to study how clients experience nature during individual outpatient psychotherapy that took place while walking in nature. More specifically we were interested in clients’ inner world experiences. All participants (*N* = 12) received treatment through licensed therapists for a DSM-5 classified disorder. Semi-structured interviews were conducted. To uncover true lived experiences during these interviews, participants were asked to close their eyes and envision themselves during a psychotherapy session in nature. The verbatim transcripts were coded by means of inductive thematic analysis and the results were member checked. Results showed that nature brings clients closer to their inner worlds. How nature brings this about is unfolded in a conceptual model of lived experience. We argue that psychotherapy can be enriched by considering nature as a supportive environment because bringing clients closer to their inner worlds is of essential value in facilitating successful treatment interventions.

## Introduction

“All social interaction is affected by the physical container in which it occurs”

Bennett and Bennett, 1970

Imagine you have an appointment with a therapist, like a psychologist or a psychiatrist. Where do you envision that you will have this appointment? Most of us visualize a room with chairs or even a sofa, with maybe a small table and a box of tissues. We automatically think of an indoor setting. How come we don’t consider having the session outdoors? The vast majority of psychotherapy treatments take place in an indoor setting (Jordan and Marshall, 2010). However, nature is considered to have restorative qualities that can potentially improve therapy success (Revell and McCloud, 2017; Cooley et al., 2020). An increasing number of initiatives are thus being developed that move psychotherapy into nature (Cooley et al., 2020). For example, people with stress related mental health disorders receive treatment in a rehabilitation garden (Adevi et al., 2018), adolescents with behavioral problems are taken on wilderness therapy camps (Fernee et al., 2019), and person-centered therapy or cognitive behavioral therapy is provided while strolling through local parks resulting in “Walk and Talk” therapy in nature (Revell and McCloud, 2017). The present paper focusses on the latter. The research aim of the present paper is to study how clients experience nature during individual outpatient psychotherapy that takes place while walking in nature. More specifically, we are interested in clients’ inner world experiences.

Psychotherapy is defined by the American Psychological Association (2012) as: “the informed and intentional application of clinical methods and interpersonal stances derived from established psychological principles for the purpose of assisting people to modify their behaviors, cognitions, emotions, and/or other personal characteristics in directions that the participants deem desirable.” Individual outpatient talk therapy is the most common way of providing evidence-based psychotherapy by licensed therapists in the western world (American Psychological Association, 2016). An equivalent for this treatment in a natural setting is: “talking therapy in natural outdoor spaces” (Cooley et al., 2020). For the present study we specified this description further by referring to individual outpatient psychotherapy while walking in nature. Even though walking is known to have restorative effects regardless of the environment (Gerber and Pühse, 2009), nature is the specific setting of interest for the present paper; because walking in nature settings is considered to have additional restorative effects compared to walking in built settings (Kondo et al., 2018; Olafsdottir et al., 2018; Twohig-Bennett and Jones, 2018). Psychotherapy in the present study is thus described as a licensed therapist and a client having their psychotherapy session while walking together outside in natural outdoor spaces. Nature can either be used in a passive manner as the décor of the session or in an active manner, as a resource for treatment interventions (Cooley et al., 2020).

Revell and McCloud (2017) already conducted a qualitative phenomenological study about therapy while walking in nature by interviewing counselors/psychotherapists instead of clients themselves and analyzing their reported observations about client experiences. The counselors/psychotherapists reported a more collaborative therapeutic relationship in nature compared to sessions indoors. Moreover, in the counselors/psychotherapists’ experience, the natural environment together with walking brings clients more in contact with their body and inner experiences. Also, in the counselors/psychotherapists’ experiences, clients have more opportunities to “express and act on their preferences and choices” in nature. For example, clients can choose a direction for the walk according to their preference. These results, however, raise the question what clients themselves experience during psychotherapy while walking in nature, because even though therapists are trained to speculate about certain intrapsychic processes, the clinicians judgment has been found to insufficiently grasp a client’s perspective and experience (Garb, 1998; Miller et al., 2015).

Dybvik et al. (2018) studied the experiences of 12 clients regarding how individuals understand the significance of nature in relation to their mental health and treatment. All participants received inpatient group therapy in a natural setting. Results of this qualitative study showed that nature fosters a closer connection to clients inner experiences, the obstacles they face, and existential meanings (Dybvik et al., 2018). Clients also reported that nature facilitates metaphors to life and human experience that can enhance the therapy process. This study already gives some insights into how clients experience nature, but focusses on clients who participated in inpatient group therapy. As outpatient individual treatment is the most common way of providing evidence-based psychotherapy by licensed therapists in the western world it is of relevance to investigate whether similar results can be found in outpatient individual treatment.

Sonntag-Öström et al. (2015) studied the experiences of 19 clients in a forest rehabilitation program. Participants were asked to regularly walk in the forest by themselves, not with a therapist. Results showed that the forest was a favorite place to rest, that it was experienced as restorative, and that the natural surroundings facilitated reflection. However, these clients went to the forest alone without a therapist, which was overwhelming for them sometimes. In their discussion the authors recommend that talks with a therapist should be added during the walks for the forest rehabilitation program, which would result in psychotherapy while walking in nature.

These exemplary studies about nature during psychotherapy (Revell and McCloud, 2017; Dybvik et al., 2018), or during rehabilitation (Sahlin et al., 2012; Sonntag-Öström et al., 2015; Poulsen et al., 2016), together with other phenomenological work on nature experiences in general (Snell and Simmonds, 2013; Naor and Mayseless, 2020; Schweitzer et al., 2021) suggest that nature can have an impact on clients’ inner world experiences. More specifically, previous work suggests that nature might facilitate coming closer to inner world experiences. Such findings have potential value for psychotherapy. Psychotherapists aim to assist their clients to modify their behaviors, cognitions, emotions and/or personal characteristics in directions that clients deem desirable. But to be able to assist their clients herein, accessibility to lived inner world experiences is required (Lang and Bradley, 2010; Thoma and McKay, 2014; Ji et al., 2016). To exemplify, when a client is convinced of her worthlessness, merely stating she is not, will not change her belief. Her therapist will want to organize a treatment intervention that will result in her experiencing that she has value. Such an experience is usually best gained when someone feels connected to their inner world, as is described in treatment interventions for numerous evidence-based treatments (Lang and Bradley, 2010; Thoma and McKay, 2014; Ji et al., 2016). If nature can be a supportive environment to foster such a connection to people’s inner worlds, it has value for psychotherapy.

The research aim of this qualitative study was to study inner world experiences regarding nature, in clients who received individual outpatient psychotherapy while walking in nature, since, to our knowledge, this has not been studied so far. Clarity and depth regarding the human-nature relationship during psychotherapy in nature might help the field of (clinical) environmental psychology by furthering our understanding of underlying psychological processes (Joye and Van den Berg, 2011; Richardson, 2019; Egner et al., 2020). Moreover, studying clients’ inner world experiences regarding nature during psychotherapy has clinical relevance, because it might help to give words to what the value of nature is and contribute to incorporating nature as a supportive environment for psychotherapy (Cooley et al., 2020).

## Materials and Methods

### Researcher Backgrounds

In line with the consolidated criteria for reporting qualitative research (COREQ; Tong et al., 2007), this section describes the researcher backgrounds and their involvement in different stages of the study process.

The lead researcher (DM) is a junior researcher (MSc) and a psychologist. She is currently working on her dissertation concerning nature and mental health. She also works as a psychologist for an outpatient department of a large mental health care facility, where psychotherapy sessions are predominantly provided indoors. DM has been involved in the present research regarding conceptualization, methodology, data collection, data analysis, and writing - original draft preparation.

NV is a senior researcher (PhD) in Nursing Science. She has ample experience in qualitative research. NV has been involved in the present research regarding methodology, data analysis, supervision, and writing – review and editing.

KD is a senior researcher (PhD) in Environmental Psychology. KD has been involved in the present research regarding conceptualization, supervision, and writing – review and editing.

LK is a professor in Clinical Neuropsychology. LK has been involved in the present research regarding supervision and writing – review and editing.

JM is a senior researcher (PhD) in Environmental Psychology. She has ample experience in investigating the association between green space and (mental) health. She recently co-edited a book titled ‘Green mental health.’ A Dutch book aimed to start a movement in which nature is used more broadly for prevention and treatment of mental health problems. JM has been involved in the present research regarding conceptualization, supervision, and writing – review and editing.

### Phenomenological Qualitative Design

A qualitative research design and phenomenology in particular was chosen because it focusses on providing a deep understanding of the lived experience of a phenomenon, in the present study being psychotherapy while walking in nature. The phenomenological research process consists of five mutually interactive and cumulative processes according to Finlay (2013, 2014): “(a) embracing the phenomenological attitude, (b) entering the lifeworld (through descriptions of experiences), (c) dwelling with horizons of implicit meanings, (d) explicating the phenomenon holistically, and (e) integrating frames of reference.”

The present study has followed these processes, meaning that:

(a) The researchers have reflected upon their own beliefs about the phenomenon intellectually as well as personally, which is known as bracketing. Bracketing is used to mitigate researcher bias due to unacknowledged preconceptions by stimulating researcher to uncover their own beliefs about a topic (Finlay, 2013, 2014; Dörfler and Stierand, 2020). Throughout the research process the researchers of this manuscript were encouraged to disclose their beliefs, both in writing before commencement of the study as well as verbally during the research group meetings by means of reflective conversations. (b) The researcher aimed to foster a rich description of participants’ experiences by using an imagery technique during which participants were asked to close their eyes and envision themselves during a psychotherapy session in nature. Questions like: “what do you see, what do you hear around you, what are you talking about, what do you feel in your body?” were asked to increase vividness and facilitate in-depth thick description of the lived experience. The aim of his imagery technique was to grasp the true lived experiences as much as possible and to not merely invite clients to answer the questions intellectually, but to resonate empathically.(c) Coding was performed by DM and NV. Consensus in the coding was reached after meticulously comparing codes from five randomly chosen interviews. Differences in interpretations were discussed until consensus was reached during coding and during discussion in the research group regarding the interpretations of the findings.

When analyzing the transcripts of the interviews, DM and NV, were open to the implicit meanings of what participants describe. Instead of approaching analysis as a merely intellectual process, the researchers aimed to truly imagine themselves in a participants’ experience to understand the essence of it. (d) When writing down the results of this phenomenological study, the researchers have used language in a manner that stayed close to the words used by the participants. Scientific rigor was facilitated by describing the step by step process of employing phenomenology as a method. To enhance transparency, direct verbatim quotes from participants as well as the researchers’ interpretations of the meaning of the phenomenon as a whole are included in the present manuscript. (e) When writing down the results, the researchers have not turned to the literature in clinical and/or environmental psychology to explain or interpret the phenomenon during the period of interviewing and analyzing. What the implications of results are for the overall research field of clinical environmental psychology is discussed in the discussion section of the present manuscript.

### Participants

All participants (*N* = 12) received treatment through licensed therapists (Dutch BIG register: 6 GZ-psychologists and one psychiatrist), who provide outpatient individual psychotherapy while walking in nature. Before commencement of the study there was no relation between the researchers and the participating therapists or their clients. Clients were mostly recruited *via* their therapists, who were contacted by means of the available information on their websites or *via* contact information provided by their colleagues. Also social media posts were put out to announce the study. Inclusion criteria for the clients were: (i) they were treated with psychotherapy while walking in nature, (ii) at the time of treatment they were suffering from a Diagnostic and Statistical Manual (DSM-5; American Psychiatric Association, 2013) recognized mental disorder, (iii) their therapist was listed in the Dutch BIG register, (iv) the client was willing to participate voluntary, and (v) the client consented to the audio recording of the interview.

### Procedure

Once a client was interested in participating, they were contacted by DM to discuss the information sheet, whereafter they signed for informed consent. The study was approved by the Research Ethics Committee (VCWE-2020-201). The semi-structured interviews were conducted in Dutch following a pre-developed topic list while maintaining room for elaboration, please see Table 1. The underlined questions were the central questions which gave most insight into the inner world experiences of clients. Participants were interviewed once by means of videoconferencing software. The interviews were audio-recorded, transcribed verbatim and anonymized. This procedure of data collection was first pilot tested with two acquaintances of DM. The interviews were focused on the embodied experience of a session of psychotherapy while walking in nature. To exemplify, when clients were asked during the interview to imagine themselves in a therapy session while walking alongside their therapist in nature again, they started to describe what they were seeing in their minds eye. This enabled the researcher to ask what role nature played in that experience; if nature was experienced to play a role and what impact that had; and what that meant for them as a person. The interviews took 45 min to 1 h. Transcripts of the interviews were member checked, which means that the participants were sent a summary of the results and were asked to what extent they recognized their own experience in the way the results were described.

**TABLE 1:** Topic list.

**1. General questions**		
• How often have you had therapy sessions while walking outside in nature?		
		∘ And how often have you had therapy sessions indoors?
• How did you get to the decision to choose a treatment where you would receive therapy while walking outside in nature?		
		∘ Or, if your therapist suggested this context for treatment, what did you think of this suggestion?
		∘ What did you expect of the sessions in nature beforehand?
• How does/did your therapist incorporate nature in the sessions?		
**2. Suggestions for questions during the Imagination**		
*Please close your eyes or look at a neutral point in front of you. Just take a few deep breaths in and out…… Try to imagine again that you are in a session, walking alongside your therapist outside in nature. Do you see it in front of you again?*		
• What do you see/hear/smell?		
• What is happening?		
• What are you discussing?		
• What are you doing?		
• What role does nature play in this experience?		
• What is going on inside of you?		
• How do you feel/how does that feel?		
• How do you notice that?		
• What do you feel in your body?		
• What does this do with you?		
• What about nature makes it so?		
• What about that moves you?		
• What does that mean for you (as a person)?		
**3. Follow-up discussion after the imagination**		
• To what extent was what you just experienced about the role of nature something typical of any session outside?		
		∘ To what extent do you think the element of nature influences what you get out of therapy?
• What would be different if this session had not happened in nature, but inside a consulting room?		

*The underlined questions were the central questions which gave most insight into the inner world experiences of clients.*

Data was collected until saturation was reached, which means that interviews from new participants were collected, transcribed verbatim and analyzed until the new interviews did not substantially reveal new information (i.e., new themes) for the description of the lived experience of the phenomenon. At this moment, two more interviews were collected and analyzed, to make sure data saturation was indeed complete.

For the purpose of publication in an international scientific journal the quotes and figures were translated into English for which a professional English speaker was involved.

### Data Analysis

Like Revell and McCloud (2017), data was analyzed by means of an inductive thematic analysis following the procedural steps of Giorgi (2009): “four systematic stages were followed: (i) transcripts were read through to gain a sense of the overall experience; (ii) in-depth re-reading of the descriptions and further reflection identified themes that were pertinent to clients experiences of psychotherapy while walking in nature. A theme statement conveyed an aspect of meaning that relates to a specific, recurring aspect of the overall experience of psychotherapy while walking in nature; and (iii) emergent themes were integrated into an exhaustive “condensation” (summary representation) that reflected client experience as a whole.” Data collection and data analysis were conducted as parallel processes with one informing the other. During this stage consensus among the researchers was strived for. To help organize this thematic analysis process, CAQDAS (Computer Assisted Qualitative Data Analysis) software was used, specifically ATLAS.ti 9.1 (Flick, 2013; Friese, 2019).

## Results

### Description of Participants

Of the twelve clients that participated in this study, eleven were referred by their therapists. One clients signed up themselves. Ten participants reported a connectedness to nature before commencement of psychotherapy while walking in nature. Two participants reported to not have experienced such a connectedness beforehand at all. Two participants reported being wary of starting sessions outside in nature, five were rather neutral beforehand and five were excited to go outdoors. In half of the treatments, the therapists took the initiative to take the therapy outside into nature; in the other half, the initiative was taken by the clients themselves. Ten participants had had previous experience with indoor psychotherapy; two participants were only familiar with nature therapy. Regarding how their therapists integrated nature in the therapy sessions, five participants reported an active use of nature or natural elements. A therapist, for example, asked a client what they saw around them and if they recognized something. However, most participants reported that therapists used nature in a passive manner, as the décor of the session. The forest was most frequently reported as the nature setting for the sessions, but dunes/beach and heathland were also mentioned. Finally, most participants had already completed treatment at the time of interview, but there were also four participants who were still in treatment. To check whether participants recognized themselves in the results a summary of the results was member checked. It had a high response rate with 11 out of 12 participants. Nearly all participants stated that they recognized themselves in the essence of the description and inconsistencies were discussed within the research team.

### Clients’ Inner Worlds Experiences Regarding Nature

**FIGURE 1:**
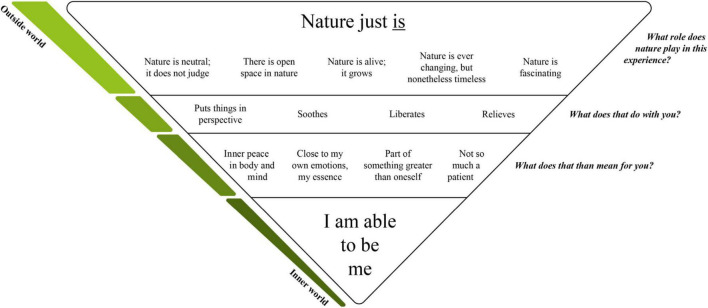
Conceptual model of experiences regarding nature during psychotherapy.

The research aim of the present paper was to study clients’ inner worlds experiences regarding nature, during psychotherapy. Inductive thematic coding revealed that experiencing the natural outside world brought clients closer to their inner worlds. We developed a conceptual model with four levels as to how nature seems to bring this about. Nearly all participants regarded nature as “nature just is” (level 1). This outside world observation had an impact on clients’ inner worlds (level 2), which amongst other things brought them closer to their emotions, for some to their essence (level 3). Deep inside, this seemed to elicit a feeling of “I am able to be me” (level 4). Please see Figure 1 for a schematic representation of this conceptual model. The four levels will be described in detail in the next four subsections.

#### Level One: “What Role Does Nature Play in This Experience?”

The first level, “nature just is,” was described in terms of one or more of five observations that were mentioned by most of the participants. These were as follows: (1) Nature is neutral and it does not judge. Participants reported experiencing nature as a neutral or accepting presence that does not judge them for their feelings, actions and who they are. Quote from participant 2: *“Yes, nature asks absolutely nothing of me, I find that one of the best experiences. Erm [short pause] yes, it’s just there and if I’m not there, it’ll keep doing the same thing [laughs], it doesn’t ask anything of me, I don’t have to adapt my behavior to it, it doesn’t judge, it doesn’t care whether I’m walking around or not, the sun will still shine. And the water flows, and not because of what I have or haven’t done. I find that a really pleasant experience, yes.”* (2) There is open space in nature: several participants reported that nature is just so vast, for example with views of an open landscape or being able to see the horizon. Quote from participant 3: “*Nature, uh, peace, uh, less oppressive, you don’t feel confined, you can see the horizon.”* (3) Nature is alive and it grows: participants reported that the experience of nature is different from experiencing other environments because nature is alive. A table is just a table and a chair is just a chair, but nature is not static, it has its own existence. Quote from participant 5: *“A leaf is a leaf and a tree is a tree. And they’ve grown, just from air and water and some soil, it’s so soothing.”* (4) Nature is ever changing, but nonetheless timeless: participants reported that nature just is, because it has always been there and/or always will be, while at the same time there is something different every day. Quote from participant 12: “*nature is there and changes with the seasons, and even if there’s a crack in it, it’s not, nothing’s really perfect so it’s actually always perfect just as it is, things just happen the way they do. It’s such a mirror and such an important lesson to be able to let things go instead of struggling so much.*” (5) Nature is fascinating: A bird that flies by or chirps, leaves that blow in the wind, waves that break on the beach; in nature there is always movement; it is quiet but never silent. Participants reported that nature is just fascinating. Quote from participant 10: *“And the same goes for a mouse running across the sand, or whatever. There’s really lots happening. Yeah, so that also has to do with those moments, there’s just loads that you automatically filter out. But you can zoom in on things infinitely. And it’s really bloody interesting.”*

#### Level Two: What Does ‘Nature Just Is’ do With You?

The five elements of “nature just is” are observations from the outside world, of what nature encompasses. Such an observation of the outside natural world had an impact on participants’ inner worlds, which constitutes the second level in the “nature just is” to “I am able to be me” experience. There was no “one on one” relationship between a particular element and a specific impact that was mentioned; several different connections were made by the participants between certain observations and the impact it had on them. A realization that “nature just is” had the following impact: (1) Puts things in perspective: most participants reported that the presence of nature puts things in perspective. For example, seeing the open space in nature or the realization of how nature is neutral provided them with some distance from their own problems. Their problems then felt smaller but not in such a way that they were trivialized or downplayed. Quote from participant 1: *“There’s no need to do anything, everything is gentle, and comes and goes*… *Hm, everything just does what it does. That tree’s just standing there happily. And that bird’s just watching a bit, there’s a few birds sitting on the ground. Yeah, they don’t worry about anything.”* (2) Soothes: several participants experienced a sense of comfort coming from nature. For example, that nature does not judge could make nature feel like a caring and reassuring presence. Quote from participant 7: *“So you can lose someone, you can actually lose someone, but you’ll never lose nature. Never. So that’s really comforting for me.”* (3) Liberates: several participants experienced how “nature just is” had a liberating impact. For example, witnessing the vastness of nature and feeling that there is so much open space could result in a liberating impact; just as experiencing no judgment from nature. Quote from participant 4: “*but the moment when you walk away afterward and when you really think, like, I’ve left it behind*… *and yeah, that’s a liberating feeling*… *in the sense that I’ve left my bit of sorrow, as it were, or mourning, or whatever you want to call it, that I’ve given it to nature*… *and that I really feel like the things that I wanted to forgive, that I’ve been able to*… *yeah, say it to that group of trees. And that they’ve listened to me and still they haven’t judged me, and that it was just the way it was.”* (4) Relieves: several participants mentioned that nature had a relieving impact, such as how nature is always changing can give hope that the worst is over; and that better times are ahead. Quote from participant 2: *“despite that I am now in that marshy swamp, where it is half drigh, half wet, half nothing, there will also be for me, there will come a time again that it is beautiful and that the sun will shine also for me.* Researcher: *and what does that than do with you, when you stand there like that and that realization comes to you?* Participant 2: *it gave me hope. Hope and relief, I think. And a sense of perspective, because this period will also pass; and perhaps also a bit of acceptance, like, yeah, that’s just how life is. One period goes and another comes. [Short pause] And even from a difficult time, something*… *new can spr-, yeah, sprout, I can’t think of another, another word at the moment. To me it means that there’s hope.”*

#### Level Three: What Does That Mean for You?

At the third level in the “nature just is” to “I am able to be me” experience, participants reported what it meant for them that “nature just is” puts things in perspective, soothes, liberates or relieves. Again, there was no “one on one” relationship between particular elements; several different connections were made by the participants between what “nature just is” did with them and what it meant for them. Regarding what it meant for them, participants described the following: (1) coming close to my emotions and essence: nearly all participants reported that it connected them to their own emotions and to what it means to be themselves or who they have become. Quote from participant 6: “*And at some point my therapist will point to a bud which hasn’t come out yet, or has just come out. It’s a nice symbol in the thorn bushes*… *that in spite of all the prickliness, there’s still something beautiful in them*… *it makes me sad, but at the same time it makes me proud too. A sort of victory feeling; the bush isn’t as prickly as it seemed at first.”* (2) Inner peace in body and mind: nearly all participants reported that what “nature just is” did with them resulted in a sense of relaxation of their bodies and a sense of calmness or peacefulness in their mind. Quote from participant 10: “…*a sort of endless falling of molecules, but none of those molecules care that they’re constantly moving, like, just bouncing around. Erm, yeah, which actually gave me a really calm feeling, it just happened. And it did matter.”* Some participants also reported that the critical meta-thinking about their own thoughts, feelings, and behavior was reduced, and hence they were more present in the moment. (3) Feeling less like a patient: several participants reported that as a result of what “nature just is” did with them, they felt less stigmatized; they felt less like a patient, because of the neutral setting and the absence of information that lets someone know that they are a ill (like a DSM-5 book). Quote from participant 11: “*That feeling of equality, that more equal feeling, like, when you’re walking in neutral surroundings, and they’re not just neutral in terms of location, because, you know, it’s not her forest, it’s not my forest, but it’s also neutral in its whole atmosphere, that’s really, erm, the difference from, from sitting indoors.”* (4) Part of something greater than oneself: several participants reported feeling a deeper connection with life and the world as a whole, as a result of the impact of “nature just is.” Instead of feeling lonesome, they felt connected with something greater than themselves. Quote from participant 5: “*Closer to my own origin, to the origin of, of humanity, millions of years ago. Of course, back then the earth was full of strange creatures but, yeah, I don’t know, it’s a, yeah, it’s a really nice feeling.”*

#### Level Four: “I am Able to Be me”

Deep inside, it meant something for them as individuals to experience the feelings of: coming closer to their emotions and essence, feeling inner peace in body and mind, feeling less like a patient, and feeling part of something greater than oneself. What this meant for them as individuals can be grasped in the profound feeling of “I am able to be me.” Quote from participant 6: *“The open landscape gives me a feeling of freedom*… *that there’s so much space. Uh-hum, you also notice that my steps get lighter, I stand up straighter and with more self-assurance, but I’m also more relaxed.”* Researcher: *“And what does it mean for you as a person to experience and feel that freedom?”* Participant 6: *“It’s really important to me. It makes me feel alive, that I’m able to be me.”*

### Walking Alongside the Professional in an Outdoor Setting

An additional finding is that participants also reported experiences regarding walking alongside their therapist in an outdoor setting and the value thereof. Walking alongside each other is not exclusive to natural settings, more specifically participants also described experiences of equality of being in a different setting with their therapist and walking alongside each other. Several participants reported feeling less like a patient while walking alongside the therapist during the session; they felt a sense of equalness with their therapist. Quote participant 9: “*I think I feel more like a patient when I am sitting across from someone, and that is less when you walk in such open space.*” They also reported experiencing that the conversation went smoothly because they did not have to face the therapist and silence was consequently less awkward. Quote participant 4: “*Well ehh, I believe that everyone knows movies in which there is sofa and a box of tissues on a table*… *in such a really awkward setting, well I just did not feel much like doing that. I also notice that, I also like to go for a walk myself; I like to be outside. When you are walking outside with your significant other or anyone really, you talk very easily. You can look around a bit and that is relaxing, thus for me it is like, I think that I have a conversation with a therapist way easier and unconstrained compared to being forced to sit across from each other.*” According to the participants these experiences resulted in being able to connect to their own feelings and with a sense of peace in body and mind. Quote from participant 8: *“and because I’m not looking directly at my therapist, but rather at nature, I think I can more easily switch from my head to my heart.”* Some participants also reported the experience that the ability to complete a walk was fulfilling. It thus seems that walking alongside their therapist also fostered a closer connection to clients’ inner worlds. It should be noted that experiences of participants regarding nature are inherently intertwined with being in a different setting with their therapist in general.

### Experienced Added Value of Psychotherapy in Nature

In addition to the research aim participants were also asked about their experiences and speculations regarding psychotherapy in nature compared to their experiences with indoor psychotherapy. Ten out of twelve participants had previous experience with indoor psychotherapy. Another additional finding is that nearly all participants felt that therapy indoors was or would have been effective for them as well. However, in their experience, psychotherapy while walking in nature had added value in the sense that it made intense emotions more manageable, or that it brought them to the essence quicker, and/or that the effect of therapy was easier to generalize to daily life because the positive effects of the sessions were to some extent associated with nature (locations) and therefore more vivid. Also part of this generalizability was being able to go to the nature locations in daily life by themselves, even when therapy is finished. Quote from participant 7: “*The fact that it’s in nature makes it feels like it’s mine. I can make it my own more; afterward, if I’m out again somewhere else, I’ve learnt to be myself, to feel. Otherwise I tend to shut myself off more after indoor sessions with my therapist. I think I can connect [the sessions in nature] to my everyday life more easily.”*

In some cases, there were circumstances in which psychotherapy while walking in nature was not perceived as an added value. For example, encountering passers-by’s was sometimes mentioned as potentially interfering. However, participants described that when actually encountering passer-by’s, they just walked by or engaged in a simple greeting, which was not experienced as interfering. Some participants did report that when feeling emotional they would rather not encounter someone, however, no instances were reported where such an interfering encounter actually occurred. The selection of the specific natural setting for the sessions was mentioned to be important with this regard. For some participants overwhelming emotions, like those experienced during trauma treatment were a reason to prefer a session behind closed doors.

## Discussion

The present qualitative research aimed to study how clients experience nature during individual outpatient psychotherapy that took place while walking in nature. More specifically we were interested in clients’ inner world experiences. Results showed that nature brought clients closer to their inner worlds. How nature brings this about was described in a conceptual model with four levels; from observations in the outside natural world to inner world experiences. Participants observed that “nature just is” (level 1) and that such a realization puts things in perspective, soothes, liberates or relieves (level 2), which on a deeper inner world level resulted in the experience of the following (level 3): it brought them close to their own emotions, for some even to their essence; made them feel lees like a patient; made them feel inner peace in body and mind; and made them feel part of something greater than themselves. What these experiences most profoundly meant for them as individuals was: “I am able to be me” (level 4). In addition, it seems that walking alongside the therapist and the natural environment both brought clients closer to their inner worlds. It might be that it is this combination that results in the experienced added value of psychotherapy while walking in nature.

The results of the present study add to previous research. Clients themselves reported how nature brought them in closer connection to their body, mind, and emotions. These findings are in line with the work of Revell and McCloud (2017) who reported that in the therapists’ experiences, therapy while walking in nature brought clients in contact with their body and inner experiences. The clients’ experiences of inner peace in body and mind in the present study relate to the experience of rest and restoration reported by Sonntag-Öström et al. (2015). The present study adds to the study of Sonntag-Öström et al. (2015) that walking with a therapist instead of alone is more representative of mainstream outpatient individual psychotherapy. The conceptual model presented in the present study of how nature fosters a closer connection to clients’ inner worlds during outpatient individual psychotherapy can be considered as a deeper understanding of the results found by Dybvik et al. (2018), because it illustrates how nature brought their clients of inpatient group therapy close to their inner experiences and existential meanings. Those clients also reported that nature facilitates metaphors to life and human experience that enhance therapy success, which was confirmed in the present study regarding how the observations of “nature just is” impacted inner world processes (Dybvik et al., 2018). Nature thus seems to foster reflection, as is also mentioned by Sonntag-Öström et al. (2015).

This study had strengths and limitations. The sample size of the present study was relatively small with 12 participants. However, data was collected until data saturation (i.e., new interviews did not reveal new themes) was reached which indicates that the results reflect the true experiences of the participants. A mental imagery technique was used to facilitate description of lived experience. To foster methodological quality and mitigate researcher bias the following measures were taken: first, bracketing was used before commencement of data collection and during the thematic inductive analysis (Hadi and Closs, 2016). Second, coding was done by two authors, of which the second author coded five interviews by random sampling of which transcripts to code. Another author also read all the transcripts to have a sense of all the data as a preparation for discussion of the interpretation of the results. The other members of the research group monitored this coding process in discussions during the research group meetings. Finally, a summary of the results was member checked with a high response rate of 11 out of 12 participants. Nearly all participants stated that they recognized themselves in essence of the description and inconsistencies were discussed within the research team.

It should be noted that all but one of the participants were recruited through their therapists. Maybe the therapists only asked clients to participate who did not have a bad nature therapy experience. If this is the case, it might have led to a somewhat overly optimistic result. However, during the interviews and member checks, participants were actively encouraged to disclose any experience regarding nature. The way the questions were asked during the interviews also enabled open answers. Two participants explicitly reported not to feel a connectedness with nature before commencement of the therapy. Thus, during the interviews participants were given ample opportunity to describe negative experiences if they had any. Nevertheless, in order to conclusively make statements about the phenomenon of psychotherapy while walking in nature, future research could explicitly include clients that had a not so positive experience or even clients that dropped out of the therapy. The present study is the first to really unfold in a conceptual model the way observations of the natural outside world impact clients’ inner worlds. Such a conceptual model of how nature is experienced could not have been discovered with a quantitative research design. The qualitative phenomenological design uniquely enabled the study of true lived inner world experiences.

### Theoretical Considerations

The present findings are in line with the two leading theories in restorative environments research, i.e., Stress-Reduction Theory (SRT) and Attention Restoration Theory (ART). According to SRT, viewing nature reduces stress by triggering a positive affective response (Ulrich et al., 1991). According to ART, natural elements engage attention in an effortless manner (soft fascination), allowing the mind to rest and replenish (Kaplan and Kaplan, 1989). Participants describe restorative experiences regarding nature during psychotherapy and hence mention an experienced added value of a natural setting compared to an indoor setting. This study adds to ART and SRT as it provides first insights into how nature is experienced and how this experience impacts the inner world of clients. The present study described the meaning that clients assign to the experience of nature. It seems that clients in the present study observed aspects in nature that resonated with the psychological distress they were struggling with. It seems they experienced a certain connectedness to nature (Capaldi et al., 2015; Lumber et al., 2018). A theoretical framework that might shed some light on this experience is Attachment Theory. Attachment theory posits that humans are bonding species (Bowlby, 1988). When distressed, people are pre-dispositioned to seek out their attachment figures for comfort and care. During childhood these attachment figures are parents or other primary caregivers. In adulthood the most important attachment figure is the romantic partner. When that attachment figure (or a mental representation thereof) is responsive, the stress system calms down and in this safe state of contentment there is a secure base from where to explore either the inner or outside world. Thus attachment theory describes ways to understand both restoration and reflection. Research already suggests that the therapeutic relationships formed by adult clients can exhibit all the essential elements of attachment bonds (Mallinckrodt, 2010; Johnson, 2019). Maybe “mother” nature can represent an attachment figure as well, especially during vulnerable times? Some initial empiric studies on nature and Attachment Theory have found inconclusive results, however, the experience of nature as an attachment figure during psychotherapy has not yet been studied (Capaldi, 2020). Future research could integrate the results of the present study to design empirical studies that test the essential characteristics of an attachment bond, defined by Mikulincer and Shaver (2007), in relation to the lived experience of nature during and after psychotherapy treatment while walking in nature. An attachment theory perspective provides a way to grasp the full lived experience of participants in the present study, where it almost seemed as if nature was perceived as an additional presence of some sorts. A presence that mirrors existence, at a moment in time when people seek ways to process through obstacles in theirs.

### Clinical Implications

A closer connection to inner world experiences has value for the practice of psychotherapy. Psychotherapists aim to assist their clients to modify the behaviors, cognitions, emotions and/or personal characteristics that they are suffering from. But to be able to assist their clients herein, accessibility to lived inner world experiences is required (Lang and Bradley, 2010; Thoma and McKay, 2014; Ji et al., 2016). A corrective positive experience is usually best gained when someone feels connected to his inner world, as is described in treatment interventions for numerous evidence based treatments (Lang and Bradley, 2010; Thoma and McKay, 2014; Ji et al., 2016). The present study also revealed that several factors about psychotherapy while walking in nature are being experienced as adding value to treatment when compared to experiences of indoor therapy. That intense emotions are experienced as more manageable in nature and that clients experience that they come to the essence quicker during psychotherapy in nature has value, because it implies that clients are less inhibited by overwhelming emotions while also being able to discuss the topics that are most essential to them. It is plausible that such processes result in a more meaningful change during treatment or in shorter treatment durations. The reported experiences that therapy was easier to generalize to daily life also has substantial value, because it might mean that clients can manage an increase in symptoms later in life effectively without having to seek out a therapist again. The conceptual model described in the present work and the experienced added value of psychotherapy while walking outside in nature have value for clinical research, because they can serve as an inspiration for measures to be used in future intervention studies.

Taken together with the broader line of research about the value of nature in psychotherapy, the present study provides an argument to implement nature in psychotherapy on a larger and more mainstream psychotherapy scale. A mainstream treatment indication method for psychotherapy that already considers more than the symptoms someone is suffering from and the therapeutic techniques that can be used to treat these symptoms is the behavioral therapeutic process of Korrelboom and ten Broeke (2014). This process considers the therapeutic context and someone’s social environment during the indication phase (and also during the treatment and evaluation phase; Korrelboom and ten Broeke, 2014). The therapeutic context covers not only the therapeutic alliance, but also the therapeutic setting (i.e., an inpatient or outpatient setting; individual contacts or group therapy). We argue that this method for treatment indication can be enriched by considering the physical environment, and more specifically nature, as part of the therapeutic context. Because like any social interaction, psychotherapy seems to be affected by the physical container in which it occurs (Bronfenbrenner, 1999; Kim et al., 2009; Jordan and Marshall, 2010).

There are some hurdles to consider, however, when considering integrating nature as a setting for psychotherapy. Cooley et al. (2020) for instance, mention implementation restrictions due to practical, therapeutic, and organizational hurdles, like workload that restricts psychologists to travel for their appointments, finding the appropriate surroundings and confidentiality issues that can occur when running into someone familiar when walking outside with your therapist (Cooley et al., 2020). These hurdles require attention from both the therapists themselves as well as from managers of larger mental health care facilities and policy makers to enable the dissemination of nature as a supportive environment for psychotherapy.

## Conclusion

The present study indicates that the natural world brings clients closer to their inner worlds. It is the first study to really unfold how nature brings this about in a conceptual model. Together with the broader line of research about the value of nature in psychotherapy, the present work underlines the importance to seriously consider nature as a supportive therapeutic environment in the indication process for mainstream psychotherapy, because evoking inner world experiences is of pivotal value in numerous evidence based treatments (Lang and Bradley, 2010; Thoma and McKay, 2014; Ji et al., 2016).

## Data Availability Statement

The raw data supporting the conclusions of this article will be made available by the authors upon request.

## Ethics Statement

The studies involving human participants were reviewed and approved by Vaste Commissie Wetenschap en Ethiek VU Amsterdam: VCWE-2020-201. The patients/participants provided their written informed consent to participate in this study.

## Author Contributions

DM was involved in the present research regarding conceptualization, methodology, data collection, data analysis, and writing—original draft preparation. NV was involved in methodology, data analysis, supervision, and writing—review and editing. KD and JM were involved in conceptualization, supervision, and writing—review and editing. LK was involved in supervision and writing—review and editing.

## Conflict of Interest

The authors declare that the research was conducted in the absence of any commercial or financial relationships that could be construed as a potential conflict of interest.

## Publisher’s Note

All claims expressed in this article are solely those of the authors and do not necessarily represent those of their affiliated organizations, or those of the publisher, the editors and the reviewers. Any product that may be evaluated in this article, or claim that may be made by its manufacturer, is not guaranteed or endorsed by the publisher.
